# Assessing the impact of novel risk loci on Alzheimer’s and Parkinson’s diseases in a Chinese Han cohort

**DOI:** 10.3389/fneur.2024.1326692

**Published:** 2024-01-31

**Authors:** Huimin Yan, Minglei Liu, Yuan Gao, Yanpeng Yuan, Xiaojing Liu, Yangyang Wang, Lanjun Li, Qingzhi Wang, Yanlin Wang, Changhe Shi, Yuming Xu, Jing Yang

**Affiliations:** ^1^Department of Neurology, The First Affiliated Hospital of Zhengzhou University, Zhengzhou, Henan, China; ^2^NHC Key Laboratory of Prevention and Treatment of Cerebrovascular Disease, Zhengzhou, Henan, China; ^3^Henan Key Laboratory of Cerebrovascular Diseases, Zhengzhou University, Zhengzhou, Henan, China; ^4^Institute of Neuroscience, Zhengzhou University, Zhengzhou, Henan, China

**Keywords:** Alzheimer’s disease, Parkinson’s disease, Parkinson’s disease dementia, single nucleotide polymorphisms, Chinese Han cohort

## Abstract

**Background:**

Overwhelming evidence points to that genetic factors contributing to the development of Alzheimer’s disease (AD) and Parkinson’s disease (PD). Genome-Wide Association Study (GWAS) has come a long way in the last decade. So far, a large number of GWAS studies have been published on neurological diseases and many other diseases, providing us with a wealth of genetic information and unique biological insights.

**Methods:**

Genomic DNA was extracted from both patients’ and controls’ peripheral blood samples utilizing the Blood Genome Extraction Kit. Single nucleotide polymorphisms (SNPs) were genotyped employing the enhanced multiple ligase detection reaction (iMLDR) technology.

**Results:**

A case-control study was conducted, involving 211 AD patients, 508 PD patients (including 117 with dementia), and 412 healthy individuals. Age and sex stratification analysis revealed that rs871269/*TNIP1* was associated with LOAD (*p* = 0.035), and rs5011436/*TMEM106B* was associated with AD in males (*p* = 0.044) in the genotype model. In the allele model, rs871269/*TNIP1* was found to be associated with PD in the Chinese Han population (*p* = 0.0035, OR 0.741, 95% CI 0.559-0.983), and rs708382/*GRN* was identified as a risk factor for Parkinson’s disease dementia (PDD) in the Chinese Han population (*p* = 0.004, odds ratio (OR) 0.354, 95% confidence interval (CI) 0.171-0.733). However, no significant associations with AD or PD were observed for the remaining four loci (rs113020870/*AGRN*, rs6891966/*HAVCR2*, rs2452170/*NTN5*, rs1761461/*LILRB2*) in terms of allele or genotype frequencies.

**Conclusion:**

This study identifies rs871269/*TNIP1* as a potential risk factor for both LOAD and PD, rs708382/*GRN* as a risk factor for PDD, and rs5011436/*TMEM106B* as associated with AD in males when stratified by age.

## 1 Introduction

Alzheimer’s disease (AD) and Parkinson’s disease (PD) rank as the first and second most common neurodegenerative diseases, respectively (1). Globally, 44 million people are living with dementia, with AD accounting for approximately 50–75 percent. Remarkably, half of these cases are from China. This number is anticipated to more than triple by 2050 due to an aging population (2). The incidence of PD increases with age, the prevalence of PD in the elderly population over 65 years old is about 1.7%, and the prevalence of PD in the elderly population over 80 years old is as high as 4%, exerting immense pressure on society (3).

Most cases of AD and PD are sporadic with unknown etiology, with both environmental and genetic factors contributing to their development (4). Overwhelming evidence points to important genetic roles (5). AD and PD may share common pathological processes, such as abnormal protein deposition. The presence of Aβ, a signature pathological protein of AD, has been reported in PD patients (6), and lewy body deposits, characteristic of PD pathology, are also found in AD cases (7). Additionally, the *APOE* and *MAPT* genes, recognized risk genes for AD (8), have also been linked to increased PD risk, highlighting potential genetic and pathological overlaps between AD and PD (9).

Cognitive dysfunction, a prevalent non-motor symptom of PD, further complicates the disease landscape. Parkinson’s disease dementia (PDD) progresses at a rate of about 10% per year in PD patients, with a cumulative prevalence of 75% in patients with PD for more than 10 years (10). The Sydney multicenter study, the *APOE* longest cohort to date, reported an 83% incidence of dementia among patients surviving more than 20 years (11), underscoring the significant overlap between PD and dementia. Thus, it is of interest to explore whether risk loci for AD may be risk factors for PD-induced dementia.

Genome-Wide Association Study (GWAS) has come a long way in the last decade (12). Researchers can detect genetic markers associated with specific traits or diseases on a genome-wide scale (13). Traditional genetic link research methods have limitations for the study of this complex trait (14), but GWAS can identify risk variants on a genome-wide scale, thus providing a new perspective for the study of the mechanism of complex diseases (15). So far, a large number of GWAS studies have been published on neurological diseases and many other diseases, providing us with a wealth of genetic information and unique biological insights (16).

A recent large-scale meta-analysis pooling 13 AD Genome-Wide Association Study (GWAS) datasets from European ancestry samples identified 38 loci, including seven loci that have not been reported previously (17). Of the 31 loci that have been reported, some researchers have verified some of them in the Chinese population (18–20). However, the relevance of these new loci to AD in Asian populations remains unexplored. In light of these findings, this study replicates AD-related genetic variants in the AD and PD population to identify additional candidate sites that might alter their risk.

## 2 Materials and methods

### 2.1 Study population

A total of 1,131 subjects of Han ethnicity were enrolled in this study, encompassing 211 AD patients, 508 sporadic PD patients, and 412 control subjects. Blood samples were collected from all participants for SNP testing, and patient information, including information related to age, sex, and education (years of schooling), was collected at the time of sampling. The simple mental state examination (MMSE) was used to screen cognitive function, and the scores of controls were in the normal range. Among PD patients, 117 were diagnosed with PDD based on the criteria proposed by the Movement Disorder Society Task Force, and we classified those with a disease course of more than 10 years and no cognitive impairment into the group of PD without dementia (PDND). All recruited patients were sporadic cases, with young-onset patients excluded from the study. Case definitions adhered to the criteria set by the National Institute on Aging–Alzheimer’s Association (2018) and Parkinson’s United Kingdom Brain Bank (1997). All study participants provided written informed consent, and the study received approval from the Ethics Committee of the First Affiliated Hospital of Zhengzhou University.

### 2.2 Genotyping and data analysis

Genomic DNA was extracted from both patients’ and controls’ peripheral blood samples utilizing the Blood Genome Extraction Kit (BioTeke Co., Beijing, China). Single nucleotide polymorphisms (SNPs) were genotyped employing the enhanced multiple ligase detection reaction (iMLDR) technology (Geneskybiotech, Shanghai, China). All pertinent specific PCR primers and ligation primers are enumerated in Supplementary Table 1.

Statistical analyses were executed using IBM SPSS Statistics 26.0. The age disparity was evaluated employing the *t*-test. The Hardy–Weinberg equilibrium in genotype frequency among controls was assessed utilizing the *χ*^2^ test. Logistic regression analysis facilitated the computation of risk analysis for each SNP in both dominant and recessive models, post adjustment for age and gender. Chi-squared tests were employed to contrast differences in sex ratio, genotype frequency, and allele frequency post-age and gender-stratified analysis. A two-tailed *p* value less than 0.05 was deemed statistically significant.

## 3 Results

We enrolled 211 AD patients, 508 sporadic PD patients, and 412 control subjects. AD and control, PD and control, and PDD and PDND had no significant difference in age and sex (Table 1). The *APOE* ε4 allele is one of the major genetic risk factors for AD (21). Unlike AD, the association between the *APOE* gene and PD is less clear. Some studies have shown an association between the *APOE* ε4 allele and the risk of PD, but the association is not as strong as it is with AD (22). Therefore, we tried to explore the relationship between *APOE*ε4(+) and PD and PDD. The results showed that *APOE*ε4(+) had significant difference between AD and control, but no significant difference between PD and control, PDD and PDND. The association between the *APOE* gene and PD still needs more research to confirm and explain.

**Table 1:** Characteristics of the study groups.

Characteristics	AD (*n* = 211)	PD (*n* = 508)	PDD (*n* = 117)	PDND (*n* = 106)	Control (*n* = 412)	*P*_1_ value	*P*_2_ value	*P*_3_ value
Age at onset, years	62.50 ± 5.09	61.36 ± 5.52	62.09 ± 4.55	60.94 ± 5.56	61.81 ± 5.20	0.115	0.444	0.091
Female, *n* (%)	133 (63.0%)	289 (56.9%)	64 (54.7%)	56 (52.8%)	248 (60.2%)	0.491	0.312	0.780
APOEε4(+), *n* (%)	62 (29.4%)	79 (15.6%)	18 (15.4%)	15 (14.2%)	68 (16.5%)	0.0002	0.695	0.796

P1, AD compared with Control; P2, PD compared with Control; and P3, PDD compared with PDND.

The frequencies of all seven variants in both case and control groups were in accordance with the Hardy–Weinberg equilibrium (*p* > 0.05). In all sites, genotype and allele frequencies of AD and control were not statistically different (Table 2). Considering that our AD population encompasses people of all ages, but these seven loci were found in the LOAD population in the study by Wightman et al. (17). In addition, the proportion of female and male patients in AD is 3:1, and female gender as a high-risk factor for Alzheimer’s disease has also been the focus of academic research in recent years (2). Therefore, we conducted a stratified analysis by age and sex. We found that rs871269/*TNIP1* was associated with LOAD (*p* = 0.035), and rs5011436/*TMEM106B* was associated with male AD (*p* = 0.044) in the genotype model (Table 3).

**Table 2:** Distribution of included SNPs in AD patients and control subjects.

SNPs	HWE (*p* value)	Association test	AD	Control	*p*	OR (95%CI)	*p* ^a^	OR (95%CI)
rs1761461	0.52	Genotypic (AA/AC/CC)	2/41/168	4/97/311				
		Dominant [(AA+AC)/CC]	43/168	101/311	0.247	0.788 (0.527–1.180)	0.242	0.775 (0.506–1.188)
		Recessive [AA/(AC + CC)]	2/209	4/408	0.978	0.976 (0.177–5.373)	0.907	0.903 (0.163–5.016)
		Alleles (A/C)	45/377	105/719	0.286	0.817 (0.564–1.184)		
rs2452170	0.87	Genotypic (GG/GA/AA)	204/7/0	393/19/0				
		Dominant [(GG + GA)/AA]	211/0	412/0	-	-	-	-
		Recessive [GG/(GA + AA)]	204/7	393/19	0.445	1.409 (0.583–3.407)	0.621	1.269 (0.494–3.257)
		Alleles (G/A)	415/7	805/19	0.450	1.399 (0.584–3.356)		
rs5011436	0.83	Genotypic (AA/AC/CC)	26/97/88	41/188/183				
		Dominant [(AA+AC)/CC]	123/88	229/183	0.518	1.177 (0.798–1.562)	0.895	1.024 (0.718–1.462)-
		Recessive [AA/(AC + CC)]	26/185	41/371	0.366	1.272 (0.754–2.144)	0.318	1.327 (0.761–2.314)
		Alleles (A/C)	149/273	270/554	0.237	1.120 (0.875–1.434)		
rs6891966	0.80	Genotypic (GG/GA/AA)	183/28/0	357/51/4				
		Dominant [(GG + GA)/AA]	211/0	408/4	-	-	-	-
		Recessive [GG/(GA + AA)]	183/28	357/55	0.978	1.007 (0.618–1.641)	0.850	1.051 (0.629–1.754)
		Alleles (G/A)	394/28	765/59	0.731	1.085 (0.681–1.729)		
rs708382	0.53	Genotypic (CC/CT/TT)	39/109/63	72/211/129				
		Dominant [(CC + CT)/TT]	148/63	283/129	0.710	1.071 (0.746–1.536)	0.995	1.001 (0.682–1.469)-
		Recessive [CC/(CT + TT)]	39/172	72/340	0.756	1.071 (0.696–1.647)	0.867	0.962 (0.611–1.513)
		Alleles (C/T)	187/235	355/469	0.678	1.051 (0.830–1.332)		
rs871269	0.56	Genotypic (CC/CT/TT)	43/98/70	67/190/155				
		Dominant [(CC + CT)/TT]	141/70	257/155	0.274	1.215 (0.857–1.722)	0.376	1.181 (0.817–1.710)-
		Recessive [CC/(CT + TT)]	43/168	67/345	0.202	1.318 (0.862–2.016)	0.116	1.439 (0.914–2.265)
		Alleles (C/T)	184/238	324/500	0.146	1.193 (0.941–1.513)		
rs113020870	-	Genotypic (CC/CT/TT)	0/0/211	0/0/412				
		Dominant [(CC + CT)/TT]	0/211	0/412	-	-	-	-
		Recessive [CC/(CT + TT)]	0/211	0/412	-	-	-	-
		Alleles (C/T)	0/422	0/824	-	-	-	

^*^A two-tailed *p* < 0.05 was considered significant.

^a^Adjusted age and sex by logistic regression.

AD, Alzheimer’s disease; SNPs, Single nucleotide polymorphisms; HWE, Hardy–Weinberg equilibrium; CI, Confidence interval; and OR, Odds ratio.

**Table 3:** Age-stratified and sex-stratified analysis of 7 loci.

SNPs	Genotype, allele	Age onset <65 years				Age onset > = 65 years				Male				Female			
		AD	Control	p	OR (95%CI)	AD	Control	*p*	OR (95%CI)	AD	Control	*p*	OR (95%CI)	AD	Control	*p*	OR (95%CI)
rs1761461	A/A	2	4	0.480		0	0	0.816		0	2	0.512		2	2	0.495	
	A/C	22	75			19	22			16	39			25	58		
	C/C	93	231			75	80			62	123			106	188		
	A	26	83	0.374	0.809 (0.506–1.292)	19	22	0.827	0.930 (0.486–1.779)	16	43	0.370	0.757 (0.412–1.392)	29	62	0.517	0.857 (0.536–1.368)
	C	208	537			169	182			140	285			237	434		
rs2452170	G/G	114	295	0.297		90	98	0.906		77	156	0.167		127	237	0.973	
	G/A	3	15			4	4			1	8			6	11		
	A/A	0	0			0	0			0	0			0	0		
	G	231	605	0.302	1.909 (0.548–6.656)	184	200	0.907	0.920 (0.227–3.732)	155	320	0.171	3.875 (0.480–31.257)	260	485	0.973	0.983 (0.359–2.688)
	A	3	15			4	4			1	8			6	11		
rs5011436	A/A	16	31	0.516		10	10	0.781		11	8	0.044^*^		15	33	0.807	
	A/C	48	139			49	49			35	79			62	109		
	C/C	53	140			35	43			32	77			56	106		
	A	80	201	0.624	1.083 (0.788–1.489)	69	69	0.551	1.134 (0.749–1.718)	57	95	0.093	1.142 (0.943–2.115)	92	175	0.848	0.970 (0.709–1.326)
	C	154	419			119	135			99	233			174	321		
rs6891966	A/A	0	2	0.668		0	2	0.225		0	1	0.782		0	3	0.431	
	A/G	15	42			13	9			10	20			18	31		
	G/G	102	266			81	91			68	143			115	214		
	A	15	46	0.610	0.855 (0.468–1.562)	13	13	0.829	1.091 (0.493–2.418)	10	22	0.902	0.953 (0.440–2.064)	18	37	0.725	0.900 (0.502–1.615)
	G	219	574			175	191			146	306			248	459		
rs708382	C/C	15	54	0.507		24	18	0.401		19	25	0.186		20	47	0.448	
	C/T	64	158			45	53			34	87			75	124		
	T/T	38	98			25	31			25	52			38	77		
	C	94	266	0.471	0.894 (0.658–1.213)	93	89	0.247	1.265 (0.850–1.883)	72	137	0.363	1.195 (0.814–1.754)	115	218	0.849	0.971 (0.719–1.312)
	T	140	354			95	115			84	191			151	278		
rs871269	C/C	22	55	0.965		21	12	0.093		13	29	0.979		30	38	0.152	
	C/T	53	141			45	49			35	72			63	118		
	T/T	42	114			28	41			30	63			40	92		
	C	97	251	0.797	1.041 (0.767–1.413)	87	73	0.035^*^	1.546 (1.031–2.318)	61	130	0.911	0.978 (0.662–1.445)	123	194	0.057	1.339 (0.991–1.809)
	T	137	369			101	131			95	198			143	302		
rs113020870	C/C	0	0	-		0	0	-		0	0	-		0	0	-	
	C/T	0	0			0	0			0	0			0	0		
	T/T	117	310			94	102			78	164			133	248		
	C	0	0	-	-	0	0	-	-	0	0	-	-	0	0	-	-
	T	234	620			188	204			156	328			266	496		

^*^A two-tailed *p* < 0.05 was considered significant.

AD, Alzheimer’s disease; SNPs, Single nucleotide polymorphisms; HWE, Hardy–Weinberg equilibrium; CI, Confidence interval; and OR, Odds ratio.

In allelic models, rs871269/*TNIP1* showed a significant difference between PD patients and controls (*p* = 0.0035, OR 0.741, 95% CI 0.559–0.983; Table 4). Similarly, a significant difference was observed in the variation of rs708382/*GRN* between PDD patients and PDND patients [*p* = 0.004, odds ratio (OR) 0.354, 95% confidence interval (CI) 0.171–0.733; Table 5], with the patient group exhibiting higher levels of the C allele compared to the control group. Because PD is divided into early-onset and late-onset according to the 40-year old boundary (23), we did not conduct a stratified analysis of PD by age and gender. Considering that we did many tests, we adopted Bonferroni correction method to adjust the statistical significance level, and the results showed that rs871269/TNIP1 was still statistically different between PD patients and controls, and rs708382/GRN was still statistically different between PDD patients and PDND controls. However, rs871269/TNIP1 and rs5011436/TMEM106B no longer showed statistical differences in AD sex and age stratification.

**Table 4:** Distribution of included SNPs in PD patients and control subjects.

SNPs	HWE (*p* value)	Association test	PD	Control	*p*	OR (95%CI)	*p* ^a^	OR (95%CI)
rs1761461	0.52	Genotypic (AA/AC/CC)	8/99/401	4/97/311				
		Dominant [(AA+AC)/CC]	107/401	101/311	0.331	0.788 (0.527–1.180)	0.488	0.775 (0.506–1.188)
		Recessive [AA/(AC + CC)]	8/500	4/408	0.533	0.976 (0.177–5.373)	0.843	0.903 (0.163–5.016)
		Alleles (A/C)	115/901	105/719	0.110	0.817 (0.564–1.184)		
rs2452170	0.87	Genotypic (GG/GA/AA)	496/11/1	393/19/0				
		Dominant [(GG + GA)/AA]	507/1	412/0	-	-	-	-
		Recessive [GG/(GA + AA)]	496/12	393/19	0.149	1.409 (0.583–3.407)	0.366	1.269 (0.494–3.257)
		Alleles (G/A)	1003/13	805/19	0.213	1.399 (0.584–3.356)		
rs5011436	0.83	Genotypic (AA/AC/CC)	56/243/208	41/188/183				
		Dominant [(AA+AC)/CC]	299/208	229/183	0.196	1.177 (0.798–1.562)	0.395	1.024 (0.718–1.462)-
		Recessive [AA/(AC + CC)]	56/451	41/371	0.967	1.272 (0.754–2.144)	0.718	1.327 (0.761–2.314)
		Alleles (A/C)	355/659	270/554	0.330	1.120 (0.875–1.434)		
rs6891966	0.80	Genotypic (GG/GA/AA)	427/81/0	357/51/4				
		Dominant [(GG + GA)/AA]	508/0	408/4	-	-	-	-
		Recessive [GG/(GA + AA)]	427/81	357/55	0.389	1.007 (0.618–1.641)	0.547	1.051 (0.629–1.754)
		Alleles (G/A)	935/81	765/59	0.621	1.085 (0.681–1.729)		
rs708382	0.53	Genotypic (CC/CT/TT)	102/253/153	72/211/129				
		Dominant [(CC + CT)/TT]	355/153	283/129	0.919	1.071 (0.746–1.536)	0.736	1.001 (0.682–1.469)-
		Recessive [CC/(CT + TT)]	102/406	72/340	0.678	1.071 (0.696–1.647)	0.812	0.962 (0.611–1.513)
		Alleles (C/T)	457/559	355/469	0.415	1.051 (0.830–1.332)		
rs871269	0.56	Genotypic (CC/CT/TT)	97/266/145	67/190/155				
		Dominant [(CC + CT)/TT]	363/145	257/155	**0.0035** ^*^	0 0.741(0.559–0.983)	**0.0047** ^*^	1.181 (0.817–1.710)-
		Recessive [CC/(CT + TT)]	97/411	67/345	0.1093	0.918(0.648–1.30)	0.116	1.439 (0.914–2.265)
		Alleles (C/T)	460/556	324/500	**0.0102** ^*^	0 0.851(0.703–1.03)		
rs113020870	-	Genotypic (CC/CT/TT)	0/0/508	0/0/412				
		Dominant [(CC + CT)/TT]	0/508	0/412	-	-	-	-
		Recessive [CC/(CT + TT)]	0/508	0/412	-	-	-	-
		Alleles (C/T)	0/1016	0/824	-	-	-	

^*^A two-tailed *p* < 0.05 was considered significant.

^a^Adjusted age and sex by logistic regression.

PD, Parkinson’s disease; SNPs, Single nucleotide polymorphisms; HWE, Hardy–Weinberg equilibrium; CI, Confidence interval; and OR, Odds ratio.

**Table 5:** Distribution of included SNPs in PDD patients and PDND patients.

SNPs	HWE (*p* value)	Association test	PDD	PDND	*p*	OR (95%CI)	*p* ^a^	OR (95%CI)
rs1761461	0.99	Genotypic (AA/AC/CC)	1/23/93	1/17/88				
		Dominant [(AA+AC)/CC]	24/93	18/88	0.501	0.793 (0.403–1.560)	0.851	1.073 (0.513–2.246)
		Recessive [AA/(AC + CC)]	1/116	1/105	0.250	3.314 (0.382–28.733)	0.944	1.110 (0.060–20.596)
		Alleles (A/C)	25/209	19/193	0.944	1.105 (0.068–17.885)		
rs2452170	0.99	Genotypic (GG/GA/AA)	115/2/0	103/3/0				
		Dominant [(GG + GA)/AA]	117/0	186/0	-	-	-	-
		Recessive [GG/(GA + AA)]	115/2	103/3	0.572	0.597 (0.098–3.644)	0.485	1.993 (0.288–13.777)
		Alleles (G/A)	232/2	209/3	0.575	0.601 (0.099–3.629)		
rs5011436	0.95	Genotypic (AA/AC/CC)	14/58/45	14/47/45				
		Dominant [(AA+AC)/CC]	72/45	61/45	0.544	0.847 (0.496–1.448)	0.598	1.171 (0.651–1.109)-
		Recessive [AA/(AC + CC)]	14/103	14/92	0.780	1.120 (0.507–2.473)	0.817	1.112 (0.453–2.732)
		Alleles (A/C)	86/148	75/137	0.763	0.942 (0.640–1.388)		
rs6891966	0.56	Genotypic (GG/GA/AA)	100/17/0	93/13/0				
		Dominant [(GG + GA)/AA]	117/0	106/0	-	-	-	-
		Recessive [GG/(GA + AA)]	100/17	93/13	0.620	1.216 (0.560–2.641)	0.456	0.720 (0.304–1.707)
		Alleles (G/A)	217/17	199/13	0.633	1.199 (0.568–2.532)		
rs708382	0.28	Genotypic (CC/CT/TT)	31/60/26	12/63/31				
		Dominant [(CC + CT)/TT]	91/26	75/31	0.230	0.691 (0.378–1.265)	0.170	1.595 (0.818–3.108)
		Recessive [CC/(CT + TT)]	31/86	12/94	**0.004** ^*^	0.354 (0.171–0.733)	**0.011** ^*^	2.801 (1.271–6.173)
		Alleles (C/T)	122/112	87/125	**0.019** ^*^	0.639 (0.439–0.930)		
rs871269	0.39	Genotypic (CC/CT/TT)	19/63/35	14/55/37				
		Dominant [(CC + CT)/TT]	82/35	69/37	0.426	0.796 (0.454–1.397)	0.498	1.236 (0.669–2.283)
		Recessive [CC/(CT + TT)]	19/98	14/92	0.524	0.785 (0.372–1.656)	0.565	1.271 (0.561–2.879)
		Alleles (C/T)	101/133	83/129	0.390	0.847 (0.580–1.237)		
rs113020870	-	Genotypic (CC/CT/TT)	0/0/117	0/0/108				
		Dominant [(CC + CT)/TT]	0/117	0/108	-	-	-	-
		Recessive [CC/(CT + TT)]	0/117	0/108	-	-	-	-
		Alleles (C/T)	0/234	0/216	-	-	-	

^*^A two-tailed *p* < 0.05 was considered significant.

^a^Adjusted age and sex by logistic regression.

PDD, Parkinson’s disease dementia; PDND, PD without dementia; SNPs, Single nucleotide polymorphisms; HWE, Hardy–Weinberg equilibrium; CI, Confidence interval; and OR, Odds ratio.

In contrast, for the remaining four sites (rs113020870/*AGRN*, rs6891966/*HAVCR2*, rs2452170/*NTN5*, and rs1761461/*LILRB2*), no statistical difference in genotype or allele frequency was noted between AD, PD, and PDD patients, and the control group. Among these risk sites, no base mutation of rs113020870 was present in any of our patients or healthy controls. Comprehensive details regarding the relationship between the seven sites and AD, PD, and PDD levels are delineated in Tables 2–5.

## 4 Discussion

Considering the impact of ethnic heterogeneity, the current study explored seven newly identified AD-related variants within the Han Chinese population. The findings revealed distinct differences in allelic modes: rs871269/*TNIP1* between LOAD patients and controls, and again rs5011436/*TMEM106B* between male AD patients and controls, rs871269/*TNIP1* between PD patients and controls, and rs708382/*GRN* between PDD patients and PDND controls. To the best of our knowledge, this research is the inaugural study to demonstrate the correlation of SNPs in *GRN*, *TNIP1*, and *TMEM106B* genes in Chinese Han Cohort. The remaining four loci exhibited no significant disparities in genotype or allele frequency between AD, PDD, PD patients, and the control group. This partial observation may be attributed to genetic heterogeneity stemming from ethnic and geographical variations, as noted by Wightman et al. (17) (refer to Supplementary Table 2 for an exhaustive overview of the seven loci of GWAS results). Moreover, the interplay between environmental and genetic factors may potentially influence gene expression. In addition, our sample size is much smaller than that of Wightman et al. (17), so our results serve as a preliminary hint.

*GRN*, a recognized frontotemporal dementia (FTD) gene (24), holds associations not only with FTD but also with LOAD (25). Its biological significance may pertain more to protein clearance mechanisms rather than involvement in specific disease-related protein aggregation. A notable pathological characteristic of PD is the presence of Lewy bodies within the cytoplasm of neurons. These are primarily composed of α-synuclein, ubiquitin, and heat shock proteins (26). Such observations suggest a potential relation of *GRN* to PD or PDD.

*TNIP1*, known to induce excessive inflammation, has been previously identified in autoimmune GWAS (27). Within mouse microglia, *TNIP1* is encompassed in a transcription module regulated by BCL3, correlating with prolonged microglial exposure to inflammation and aging (28). Various pieces of evidence have underscored that α-synuclein is the pivotal trigger of PD neuroinflammation (29). Some studies have discerned that α-synuclein can mediate the oxidative stress of microglial cells (30). However, the assertion that neuroinflammation is a fundamental process of PD remains a topic of uncertainty. The specific role of *TNIP1* in the pathogenesis of PD necessitates further exploration.

*TMEM106B* has been previously linked with dementia, albeit not in preceding LOAD GWAS (31). The rs5011436 is an intronic variant of *TMEM106B*. The proximate exon variant, rs3173615, is postulated to be an associated signal-driven variant in FTD, leading to a diminished abundance of transmembrane protein 106B (the protein encoded by *TMEM106B*) through augmented protein degradation (32). Noteworthy differences in the expression of *TMEM106B* in extensive brain tissues of LOAD patients compared with the control group have been observed (33). The intricate relationship of *TMEM106B* with AD warrants further comprehensive research.

The present study harbors certain limitations, including a relatively modest sample size. After the Bonferroni correction, rs871269/TNIP1 and rs5011436/TMEM106B no longer showed statistical differences in AD sex and age stratification. Although Bonferroni correction is relatively strict, it still needs our further attention and verification. Interestingly, in Wightman et al. (17), rs871269 T allele is associated with lower AD risk, and our study indicates a protective effect in PD. However, the T allele is the dominant allele in our study population, while it is the minor allele in the European population, considering that it may be due to racial differences. It is imperative to note that the molecular mechanisms underlying the associations between rs708382/*GRN* and rs871269/*TNIP1* and PD remain enigmatic. The design of additional functional experiments is essential to further elucidate the pathogenesis.

In conclusion, our research indicates that the variation of *TNIP1* is significantly associated with LOAD and PD within the Chinese Han population, while *TMEM106B* with male AD and *GRN* is correlated with PDD. The involvement of geographic or environmental factors in the genetic outcomes at these sites remains an open question. Comprehensive further genetic analyses and functional studies are imperative to unravel the roles of these variants in the pathogenesis of AD and PD.

## Data availability statement

The original contributions presented in the study are included in the article/Supplementary material; further inquiries can be directed to the corresponding authors.

## Ethics statement

The study received approval from the Ethics Committee of the First Affiliated Hospital of Zhengzhou University. All study participants provided written informed consent.

## Author contributions

HY: Conceptualization, Formal analysis, Methodology, Software, Writing – original draft, Validation, Writing – review & editing. ML: Formal analysis, Methodology, Writing – original draft. YG: Writing – original draft, Conceptualization, Data curation, Supervision. YY: Conceptualization, Supervision, Validation, Writing – original draft. XL: Conceptualization, Supervision, Validation, Writing – original draft. YangW: Writing – review & editing. LL: Writing – review & editing. QW: Writing – review & editing. YanlW: Writing – review & editing. CS: Writing – review & editing. YX: Funding acquisition, Writing – review & editing. JY: Formal analysis, Funding acquisition, Writing – review & editing.
